# Association and Genetic Identification of Loci for Four Fruit Traits in Tomato Using InDel Markers

**DOI:** 10.3389/fpls.2017.01269

**Published:** 2017-07-19

**Authors:** Xiaoxi Liu, Xiaolin Geng, Hongchi Zhang, Huolin Shen, Wencai Yang

**Affiliations:** Beijing Key Laboratory of Growth and Developmental Regulation for Protected Vegetable Crops, Department of Vegetable Science, China Agricultural University Beijing, China

**Keywords:** *Solanum lycopersicum*, fruit traits, association analysis, linkage disequilibrium, InDel markers

## Abstract

Tomato (*Solanum lycopersicum*) fruit weight (FW), soluble solid content (SSC), fruit shape and fruit color are crucial for yield, quality and consumer acceptability. In this study, a 192 accessions tomato association panel comprising a mixture of wild species, cherry tomato, landraces, and modern varieties collected worldwide was genotyped with 547 InDel markers evenly distributed on 12 chromosomes and scored for FW, SSC, fruit shape index (FSI), and color parameters over 2 years with three replications each year. The association panel was sorted into two subpopulations. Linkage disequilibrium ranged from 3.0 to 47.2 Mb across 12 chromosomes. A set of 102 markers significantly (*p* < 1.19–1.30 × 10^−4^) associated with SSC, FW, fruit shape, and fruit color was identified on 11 of the 12 chromosomes using a mixed linear model. The associations were compared with the known gene/QTLs for the same traits. Genetic analysis using F_2_ populations detected 14 and 4 markers significantly (*p* < 0.05) associated with SSC and FW, respectively. Some loci were commonly detected by both association and linkage analysis. Particularly, one novel locus for FW on chromosome 4 detected by association analysis was also identified in F_2_ populations. The results demonstrated that association mapping using limited number of InDel markers and a relatively small population could not only complement and enhance previous QTL information, but also identify novel loci for marker-assisted selection of fruit traits in tomato.

## Introduction

Tomato (*Solanum lycopersicum*) is one of the most consumed vegetables and ranks second in production among the solanaceous crops worldwide (http://www.fao.org/statistics/en/). It is also an excellent plant genetic analysis system, particularly for investigating the mechanisms of fruit development, color formation, and nutrient accumulation. Furthermore, clinical nutrition studies have suggested that increased consumption of tomato products can improve human health and reduce the risk of developing certain cancers (Giovannucci et al., 2002; Etminan et al., 2004; Burton-Freeman and Sesso, 2014; Perveen et al., 2015; Pourahmadi et al., 2015; Raiola et al., 2015). The combination of the economic importance and the potential health benefits make tomato fruits an important target for increasing the nutritional properties.

It has been well-known that wild tomato species bear small, round, red, or green fruits (Alpert et al., 1995). These fruits usually contain high concentration of nutritional properties such as lycopene (Hyman et al., 2004; Kinkade and Foolad, 2013) and soluble solid content (SSC; Rick, 1974; Osborn et al., 1987). Domestication and breeding alternating fruit characters result in a wide diversity of fruit color, weight, and shape (Grandillo et al., 1999; Bai and Lindhout, 2007). Unfortunately, the nutritional properties are usually lower in cultivated varieties than in wild species due to the increase of fruit size (Markovic et al., 1997; Hyman et al., 2004). Many studies have been carried out to identify genes/QTLs for fruit traits using structural populations (e.g., F_2_ population, backcross population, advanced backcross population) derived from crosses between cultivated varieties and wild species. This approach maximizes the marker polymorphisms and has led to the discovery of new genes. To date, many loci for weight (FW), shape, color, and SSC of tomato fruits have been reported from various species using this classical genetic analysis approach, and some of them have been validated using different mapping populations (Chen et al., 1999; Grandillo et al., 1999; Liu et al., 2003; Ashrafi et al., 2012; Zhou et al., 2016). However, only two loci for fruit weight (*fw2.2* and *fw3.2*), four loci for fruit shape or size (*ovate, sun, lc*, and *fas*), three loci for SSC (*LIN5, sucr*, and *Agp-L1*), and several loci for color (e.g., *r, gf*, *og*^*c*^, *t, y, hp1, hp2, hp3*, and *del*) have been well-characterized (Chetelat et al., 1995; Liu et al., 2003; Fridman et al., 2004; Petreikov et al., 2009; The Tomato Genome Consortium, 2012; van der Knaap et al., 2014).

Association analysis represents an alternative to bi-parental linkage mapping for the determination of the genetic basis of traits by allowing evaluation of a large number of alleles in diverse populations, which provides high mapping resolution and reduction in time to develop a mapping population (Rafalski, 2010; Cericola et al., 2014; Pace et al., 2015). Several attempts have been performed to identify loci for fruit traits using association analysis in tomato. These attempts can be classified into three strategies based on the techniques used for genotyping. The first strategy is re-sequencing the whole genomes of certain number of tomato accessions to conduct genome-wide association analysis, resulting in identification of the well-characterized *fas* gene for fruit size (Shirasawa et al., 2013), *fw2.2* locus for fruit weight and *y* gene for pink fruit (Lin et al., 2014), and 251 association signals for flavor chemicals in tomato fruit (Tieman et al., 2017). The second strategy is to run large-scale genotyping of various collections of tomato accessions using the tomato array platform of Solanaceae Coordinated Agricultural Project (SolCAP) or Centre of BioSystems Genomics (CBSG) for association analysis. This approach has identified phenotype/genotype associations for over 20 fruit traits including fruit color, FW, SSC, and fruit shape (Ruggieri et al., 2014; Sauvage et al., 2014; Sacco et al., 2015; Bauchet et al., 2017). The third strategy is to genotype a collection of tomato accessions with a relatively small amount of markers for association, which has detected hundreds of genotype/phenotype associations for fruit traits (Xu et al., 2013; Zhang et al., 2015, 2016; Zhao et al., 2016). All these efforts suggest that association mapping can be used to identify loci conferring agricultural traits in tomato. However, except for the re-sequencing approach, only few loci have been connected to known loci in other association studies. There is no published report of using marker-trait associations discovered through association mapping in fruit quality improvement programs for the purpose of marker-assisted selection in tomato.

Due to the abundance and wide distribution of single nucleotide polymorphisms (SNPs) in the whole genome and the availability of automatic large-scale genotyping platform, SNPs have been popularly used in association analysis in tomato (Robbins et al., 2011; Shirasawa et al., 2013; Lin et al., 2014; Ruggieri et al., 2014; Sauvage et al., 2014; Sacco et al., 2015; Sim et al., 2015; Bauchet et al., 2017). However, as the second abundant form of genetic variation in the whole genome (Yang et al., 2014), InDel markers have not been widely used in genetic study. Particularly, the use of limited number of InDel markers along with a relatively small population has not been tested in association analysis. The present study used a strategy of combining association mapping and classical genetic analysis to identify loci for four fruit traits including FW, SSC, fruit shape, and color. The cost-effective InDel markers were used to genotype a diverse collection of 192 tomato accessions consisted of *S. lycopersicum, S. lycopersicum* var. *cerasiforme*, and *Solanum pimpinellifolium*. The experience gained here will help refine strategies for genome-wide identification of quantitative loci conferring traits with economic importance in tomato and other species at an affordable level.

## Materials and methods

### Plant materials and experimental design

The association mapping panel consisted of 192 tomato accessions including 10 of *S. pimpinellifolium*, 18 of *S. lycopersicum* var. *cerasiforme* or cherry tomato, and 164 of *S. lycopersicum*, which were obtained from various sources (Table S1). The 164 *S. lycopersicum* accessions included 23 vintage varieties, 20 Latin American cultivars, 54 fresh-market lines, 59 processing lines, and 8 lines with unknown type. The association panel was grown in a randomized complete block design with three blocks containing each accession in two independent experiments conducted in 2013 and 2014. Plots of each accession consisted of at least four plants.

To identify loci for SSC and FW using the structural population mapping approach, two F_2_ populations were developed by crossing processing tomato varieties OH88119 and OH9242, respectively, to a cherry tomato line Black cherry. Both OH88119 and OH9242 have medium-sized fruit (average FW 48.3 g for OH88119 and 73.7 g for OH9242 in 2013 and 2014) with relatively low SSC (average 4.1% for OH88119 and 4.6% for OH9242 in 2013 and 2014), while Black cherry is a small-fruited tomato line (average FW 18.6 g in 2013 and 2014) with a relatively high SSC (average 6.3% in 2013 and 2014). A sub-population of 503 individuals from the F_2_ population of OH88119 × Black cherry were grown in the fall season of 2012, and another sub-population consisting of 752 individuals from the same cross as well as 276 individuals from the F_2_ population of OH 9242 × Black cherry were grown in the spring season of 2013.

Tomato seeds for all studies were sown in 288 cell flats filled with a mixture of peat and vermiculite (3:1) in a protected greenhouse. Seedlings were transplanted ~50 days after germination to field. All experiments were conducted at Shangzhuang Research Station of China Agricultural University in Beijing, China. Production practices, plant spacing, and row spacing were as recommended for commercial growers (Gao et al., 2010).

### Phenotypic data collection and analysis

Phenotypic data for association analysis were collected on a plot basis. Five to ten ripe fruits were harvested from each plot and subjected to SSC, FW, fruit height, fruit width, and color measurements.

Total weight of fruits collected in each plot was obtained by weighing all fruits using a pair of balances. Mean was obtained by dividing the total FW by the number of fruits and used as FW for individual fruit in each plot. The maximum height and width of a fruit were measured using a vernier caliper (Hangzhou Tool and Measuring Tool Company, Hangzhou, China). The fruit shape index (FSI) was calculated as the ratio of maximum height to maximum width (Brewer et al., 2006). Numeric descriptions of the red, green, yellow, and blue components of tomato color were obtained using the software Tomato Analyzer 3.0 (Brewer et al., 2006) following the description in Darrigues et al. (2008). The software generated a set of L^*^, a^*^, b^*^, hue, and chroma values representing absolute color for each fruit. SSC was measured using a WAY-2S digital ABBE refractometer (Shanghai Precision Scientific Instrument Company, Shanghai, China). Plot means for FSI, values of color parameters and SSC were calculated based on measurements of all fruits in each plot. Pearson correlation coefficients for each trait between 2 years and among traits were obtained using SAS v9.4 (SAS Institute, Cary NC, USA). Analysis of variance was conducted using PROC GLM in SAS with the model show to best fit the data: *X*_*ijb*_ = μ + *G*_*i*_ + *Y*_*j*_ + *R*_*b*/*j*_ + *M*_*ij*_+ ε_*ijb*_, where *X*_*ijb*_ is the trait value of the *b*^*th*^ replication of the *i*^*th*^ genotype in the *j*^*th*^ year, *G*_*i*_ is the random effect of the *i*^*th*^ genotype, *Y*_*j*_ represents the fixed effect of the *j*^*th*^ year, *R*_*b*/*j*_ is the fixed effect of the *b*^*th*^ replication in the *j*^*th*^ year, *M*_*ij*_ the random effect of the genotype by year interaction and ε_*ijb*_ is the residual. Broad sense heritability (*H*^2^) for each trait was calculated based on the plot level using the equation *H*^2^ = σ_*G*_^2^/(σ_*G*_^2^ +σ_*GY*_^2^ +σ_ε_^2^) according to the description in Nyquist and Baker (1991), where σ_*G*_^2^ is genotypic variance, σ_*GY*_^2^ is the variance due to interaction between genotype and year, andσ_ε_^2^ is the error variance.

### Marker analysis

A total of 547 InDel markers (Table S2) evenly distributed across the tomato genome were used to genotype the association panel. These InDel markers were chosen from our previous study and were polymorphic within 10 accessions of *S. lycopersicum* (Yang et al., 2014). Genomic DNA was isolated from fresh-collected young leaves of each accession using the modified CTAB method (Kabelka et al., 2002). PCR and genotypic data collection were conducted according to the description in Yang et al. (2014).

Nei's genetic distance (Nei, 1972) was calculated for each pair of accessions and marker allele frequency was obtained using the software PowerMarker V3.25 (Liu and Muse, 2005). Polymorphism information content (PIC) was calculated using the formula (Weir, 1990) of PIC = 1-Σ*p*_*i*_^2^, where *p*_*i*_ is the frequency of *i*^th^ allele for each marker locus. Markers with a minor allele frequency below 5% were removed from the marker data set to calculate population structure, kinship, and to perform association analysis.

### Linkage disequilibrium analysis

Marker genotypes were used to measure the extent of linkage disequilibrium (LD) within the 164 accessions of *S. lycopersicum* and 18 accessions of *S. lycopersicum* var. *cerasiforme*. The TASSEL 2.1 (Bradbury et al., 2007) software was used to calculate pair-wise *r*^2^-values for markers polymorphic within the 164 accessions. *P-*values for each *r*^2^ estimate were calculated using 1,000 permutations in TASSEL.

LD decay was calculated by plotting pair-wise *r*^2^-values onto genetic distance in base pairs on the same WGS chromosome (SL2.40) of tomato variety Heinz1706 (The Tomato Genome Consortium, 2012). All markers with <25% missing data and a minor allele frequency >5% were used to calculate LD decay. Critical values of *r*^2^ as an evidence of linkage were derived from the parametric 95th percentile of the distribution of the unlinked markers (Breseghello and Sorrells, 2006).

### Population structure and association analysis

Population structure (Q matrix) was estimated using Structure 2.3.4 software (Pritchard et al., 2000; Falush et al., 2003, 2007). Number of populations (*K*) was determined following the instruction in Pritchard et al. (2000) with a burn-in period of 100,000 iterations and Markov Chain Monte Carlo of 100,000. Twenty independent runs were done for *K* varying from 1 to 10. The most probable *K-*value was defined according to the method proposed by Evanno et al. (2005).

Unweighted Pair Group Method with Arithmetic Mean (UPGMA) cluster analysis was performed to develop a phylogenetic tree using the software PowerMarker V3.25 and the tree was viewed in MEGA5 (Tamura et al., 2011). Principal coordinate analysis (PCoA) was conducted using the Past 3.13 software (Hammer et al., 2001). The Loiselle kinship coefficients between tomato lines (*K* matrix) were calculated using the software SpAGeDi (Hardy and Vekemans, 2002).

The software program TASSEL 2.1 was used to conduct association analysis. A mixed linear model (MLM) taking into account both population structure (Q matrix) and the kinship matrix (*K* matrix), and a general linear model (GLM) using population structure (Q matrix) as a fixed factor were used for association identification of loci conferring fruit traits. Significance of marker-trait association was determined based on *p*-value at a level of 5% after Bonferroni (1936) multiple test correction. Since it has been popularly proved that MLM+Q+*K* model is a more effective approach than other models for detecting loci (Yu et al., 2006; Malosetti et al., 2007; Cericola et al., 2014; Pace et al., 2015; Sim et al., 2015), only the data of the MLM+Q+*K* model was presented in the current study. The phenotypic variation explained by each marker was the *R*^2^-value obtained from GLM model.

### Linkage analysis of loci for FW and SSC using F_2_ populations

Since most markers significantly associated with loci for FW or SSC were not polymorphic between Black cherry and OH88119 or OH9242, we selected InDel markers that showing polymorphisms between the parents in Yang et al. (2014). Our goal was to identify two markers per chromosome arm in order to survey the whole genome. Thus, a total of 56 additional markers (Table S3) distributed on 12 chromosomes were used for initial identification of loci conferring FW and SSC in the F_2_ population of OH 88119 × Black cherry. ANOVA using SAS v9.4 with a general linear model *X*_*i*_ = μ + *M*_*i*_+ ε_*i*_ (Yang et al., 2005) was performed to determine an association between trait and marker genotype of 126 individuals randomly picked from the F_2_ population of OH88119 × Black cherry. Once a marker was identified to be significantly (*P* < 0.05) associated with a trait, the marker was used to genotype the whole F_2_ population of OH88119 × Black cherry and the F_2_ population of OH9242 × Black cherry for validation.

## Results

### Marker polymorphisms

**Figure 1:**
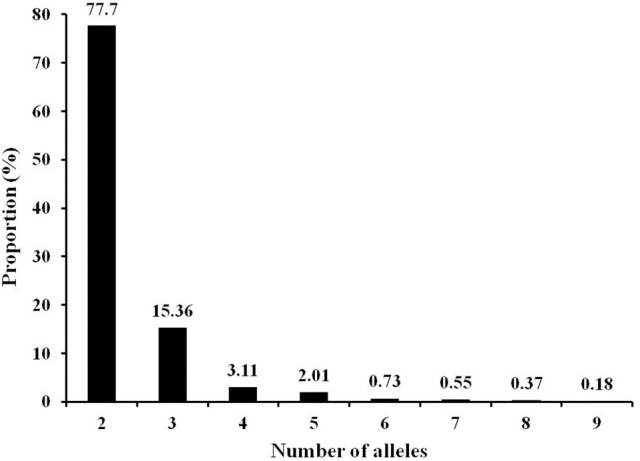
Frequency distribution of alleles in 192 tomato accessions generated by 547 InDel markers.

The 547 markers generated 1295 alleles in the 192 tomato accession with a range of two to nine alleles (average 2.4 alleles) for individual markers (Table S2). Among the polymorphic markers, ~93% had two or three alleles with the dominance of bi-allele markers (Figure 1). As expected, all markers were polymorphic in the 192 accessions. However, polymorphisms within species was decreased from wild species *S. pimpinellifolium* to *S. lycopersicum* var. *cerasiforme* and then to *S. lycopersicum*, the cultivated tomato. The proportion of polymorphic markers were 97.4% in 10 accessions of *S. pimpinellifolium*, 85.2% in 18 accessions of *S. lycopersicum* var. *cerasiforme*, 64.7% in 23 vintage accessions, 78.4% in 20 Latin American Cultivars, 67.3% in 54 fresh-market lines, and 73.3% in 59 processing lines (Table S2). Subsequently, the average polymorphic information content (PIC) also decreased from wild species *S. pimpinellifolium* to *S. lycopersicum* var. *cerasiforme* and then to *S. lycopersicum* (Table S2).

### Phenotypic variation

Significant difference for each trait was observed among genotypes, and FSI was the only trait that showed no significant difference between 2 years or three replications (Table S4). However, the phenotypic data for each trait in 2 years were highly correlated with the correlation coefficient (*r*) of 0.85 for SSC, 0.91 for FW, 0.96 for FSI, 0.86 for L^*^, 0.92 for Hue, and 0.83 for Chroma. Thus, the mean of the 2-year data for each trait was used for the following analysis.

A wide range of variation was observed for SSC, FW, FSI, L^*^, Hue, and Chroma in the 192 tomato accessions (Table 1, Figure S1). The average of SSC was higher in *S. pimpinellifolium* than in *S. lycopersicum*, while the mean of FW was smaller in *S. pimpinellifolium* than in *S. lycopersicum*, and the means of SSC and FW in *S. lycopersicum* var. *cerasiforme* were between those in *S. pimpinellifolium* and *S. lycopersicum*. Significant negative correlation was observed between SSC and FW (*r* = −0.43, *p* < 0.0001). No differences of FSI were observed among three species. The means for fruit color parameters L^*^, Hue, and Chroma were lower in *S. pimpinellifolium* than in *S. lycopersicum* var. *cerasiforme* and *S. lycopersicum*. Broad-sense heritability for each trait was obviously different. Chorma was the least heritable trait with heritability of 0.69, while FSI was the most heritable trait with heritability of 0.91 (Table 1).

**Table 1:** Range, mean, standard deviations (SD), coefficients of variation (CV) and broad-sense heritabilities (*H*^2^) collected for all traits in 192 tomato accessions.

**Trait**	**Code**	**Species**	**Minimum**	**Maximum**	**Mean**	***SD***	**CV**	***H^2^***
Soluble solid content (%)	SSC	Combined	2.84	7.67	4.43	0.83	0.19	0.71
		*Solanum lycopersicum*	2.84	5.55	4.22	0.48	0.11	
		*Solanum lycopersicum* var. *cerasiforme*	3.55	6.73	5.3	1.01	0.19	
		*Solanum pimpinellifolium*	4.93	7.67	6.90	0.90	0.13	
Fruit weight (g)	FW	Combined	0.78	311.16	91.98	56.57	0.62	0.80
		*Solanum lycopersicum*	15.57	311.16	105.38	49.85	0.47	
		*Solanum lycopersicum* var. *cerasiforme*	2.04	41.10	17.92	11.67	0.65	
		*Solanum pimpinellifolium*	0.78	2.60	1.31	0.57	0.43	
Fruit shape index	FSI	Combined	0.64	2.19	1.02	0.27	0.26	0.91
		*Solanum lycopersicum*	0.64	2.19	1.02	0.27	0.27	
		*Solanum lycopersicum* var. *cerasiforme*	0.86	1.71	1.14	0.26	0.23	
		*Solanum pimpinellifolium*	0.95	1.10	1.00	0.05	0.05	
Color—darkness or lightness	L^*^	Combined	26.12	68.23	48.43	6.82	0.14	0.75
		*Solanum lycopersicum*	35.66	68.23	49.11	5.60	0.11	
		*Solanum lycopersicum* var. *cerasiforme*	26.12	67.65	47.66	11.49	0.24	
		*Solanum pimpinellifolium*	33.89	58.97	38.86	8.26	0.21	
Color—basic tint	Hue	Combined	37.69	99.87	55.66	14.13	0.26	0.84
		*Solanum lycopersicum*	40.12	99.87	53.51	11.69	0.22	
		*Solanum lycopersicum* var. *cerasiforme*	43.58	95.42	68.81	22.95	0.33	
		*Solanum pimpinellifolium*	37.69	94.62	49.51	18.86	0.38	
Color—saturation or vividness	Chroma	Combined	25.99	55.68	45.46	4.54	0.10	0.69
		*Solanum lycopersicum*	33.55	55.68	46.03	4.31	0.09	
		*Solanum lycopersicum* var. *cerasiforme*	25.99	50.09	42.25	5.49	0.13	
		*Solanum pimpinellifolium*	37.95	44.09	41.61	2.08	0.05	

### Population structure

**Figure 2:**
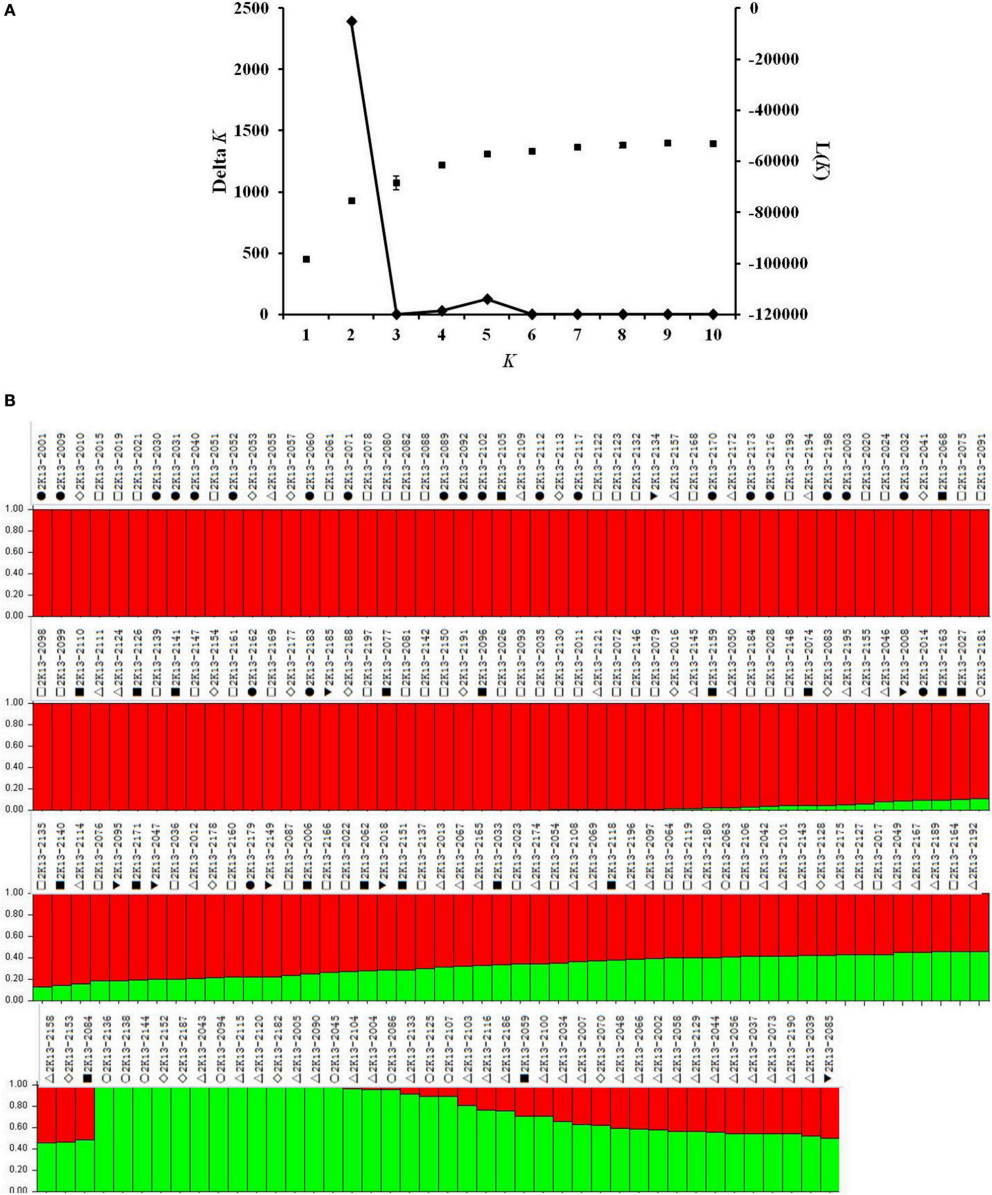
**(A)** L(*K*) and Δ*K* plots derived from the InDel data. Δ*K* is calculated as the mean of the absolute values of *L”*(*K*) averaged over 20 runs divided by the standard deviation of *L*(*K*). Δ*K* = *m*(|*L”*(*K*)|)/*s*[*L*(*K*)], which expands to Δ*K* = *m*(|*L*(*K*+1)-2*L*(*K*)+*L*(*K*-1)|)/*s*[*L*(*K*)]. *L*(*K*) is the Pr(*X*|*K*) referred as “Ln P(D)” in the output of STRUCTURE software. **(B)** Population structure estimates based on 547 InDel markers distributed across the tomato genome. The area of two different colors (Red and Green) illustrates the proportion of each subpopulation based on these InDel markers. Symbols represent accessions from different species or market class of tomato: ○ *Solanum pimpinellifolium*, ◊ *S. lycopersicum* var. *cerasiforme* or cherry tomato, ■ Latin American Cultivar, □ Fresh-market, Δ Processing, • Vintage, ▼ Unknown type. The accession of each plot number can be found in Table S1.

**Figure 3:**
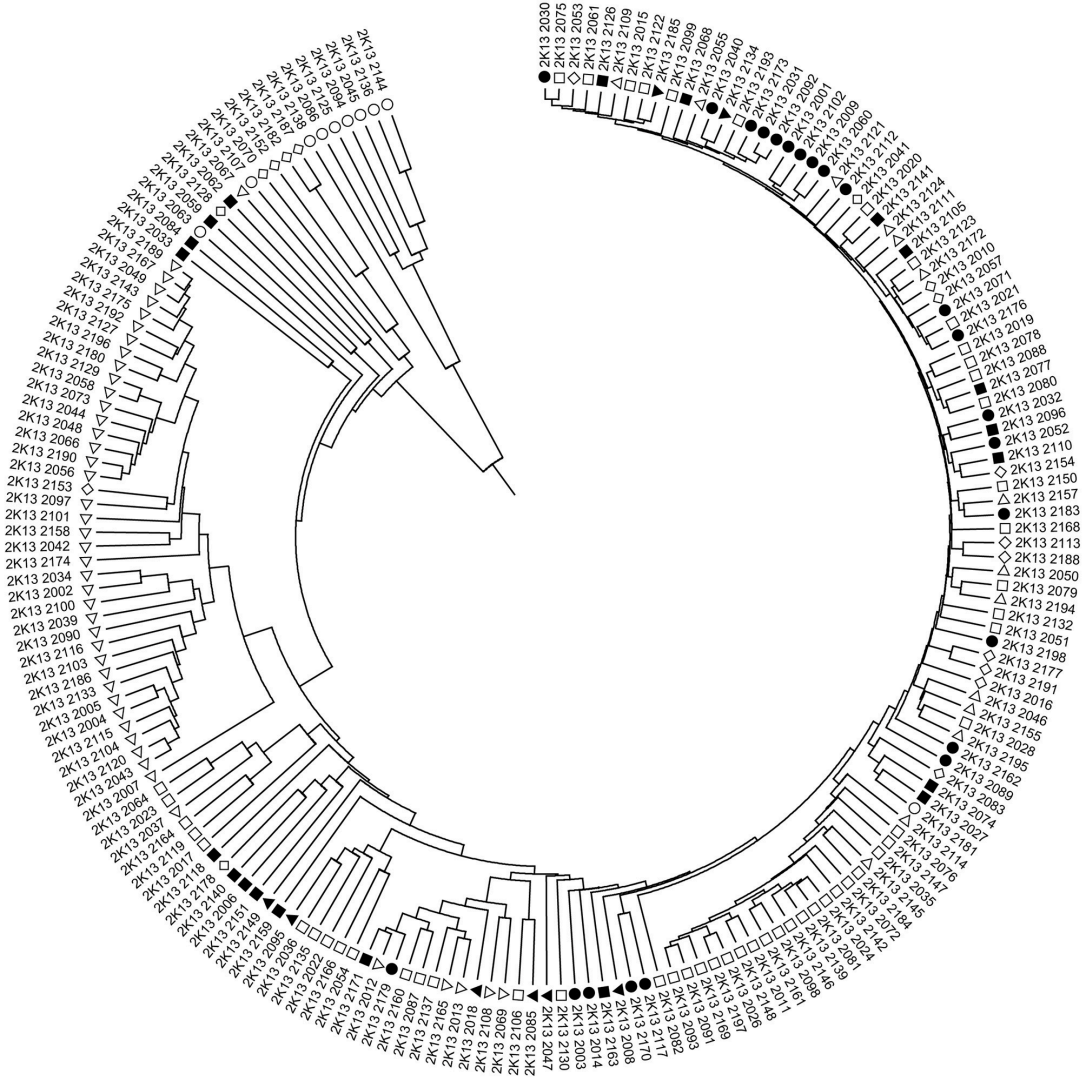
UPGMA dendrogram of 192 tomato accessions based on 547 InDel marker data. Symbols represent accessions from different species or market class of tomato: ○ *Solanum pimpinellifolium*, ◊ *S. lycopersicum* var. *cerasiforme* or cherry tomato, ■ Latin American Cultivar, □ Fresh-market, Δ Processing, • Vintage, ▼ Unknown type. The accession of each plot number can be found in Table S1.

**Figure 4:**
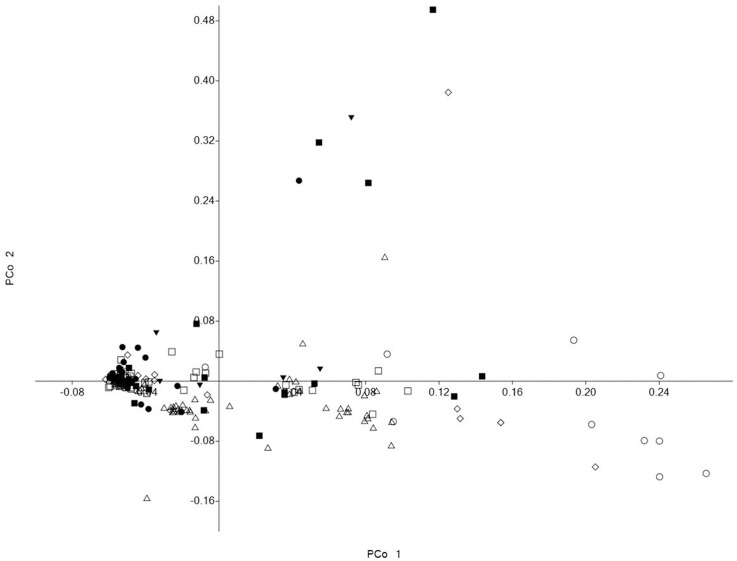
PCoA visualization of the genetic relationships between members of the association panel. Symbols represent accessions from different species or market class of tomato: ◊ *Solanum pimpinellifolium*, ○ *S. lycopersicum* var. *cerasiforme* or cherry tomato, ■ Latin American Cultivar, □ Fresh-market, Δ Processing, • Vintage, ▼ Unknown type.

Although, the 192 tomato accessions were from three species, model without prior population information was used to assign individual accession to a subpopulation using the software package of STRUCTURE2.3.4. In order to define the number of subpopulations within the 192 accessions, a series of independent runs of the data were run at a range of *K*-values from 1 to 10. The summary plot of membership coefficients (Q) and Δ*K* analysis (Figure 2) from STRUCTURE software, and the genetic relationships revealed by the phylogenetic analysis (Figure 3) and the PCoA (Figure 4), suggested that the mapping population was sorted into two subpopulations (*K* = 2). The larger subpopulation was composed of 134 accessions including all fresh-market, vintage, and unknown type accessions. One accession of *S. pimpinellifolium* LA2183, two-thirds of *S. lycopersicum* var. *cerasiforme* or cherry tomato, one-third of processing accessions, and four-fifths of Latin American Cultivars were also assigned into the larger subpopulation. Although, there was no certain trend of clustering in the larger subpopulation, the fresh-market accessions from Florida, USA formed one independent cluster. The smaller subpopulation consisted of 58 accessions including nine accessions of *S. pimpinellifolium*, two-thirds of processing, one-third of *S. lycopersicum* var. *cerasiforme* or cherry tomato, and four Latin American Cultivars. Within the smaller subpopulation, 38 processing accessions were clustered together, while the seven accessions of *S. pimpinellifolium* formed one cluster.

### Linkage disequilibrium evaluation

**Figure 5:**
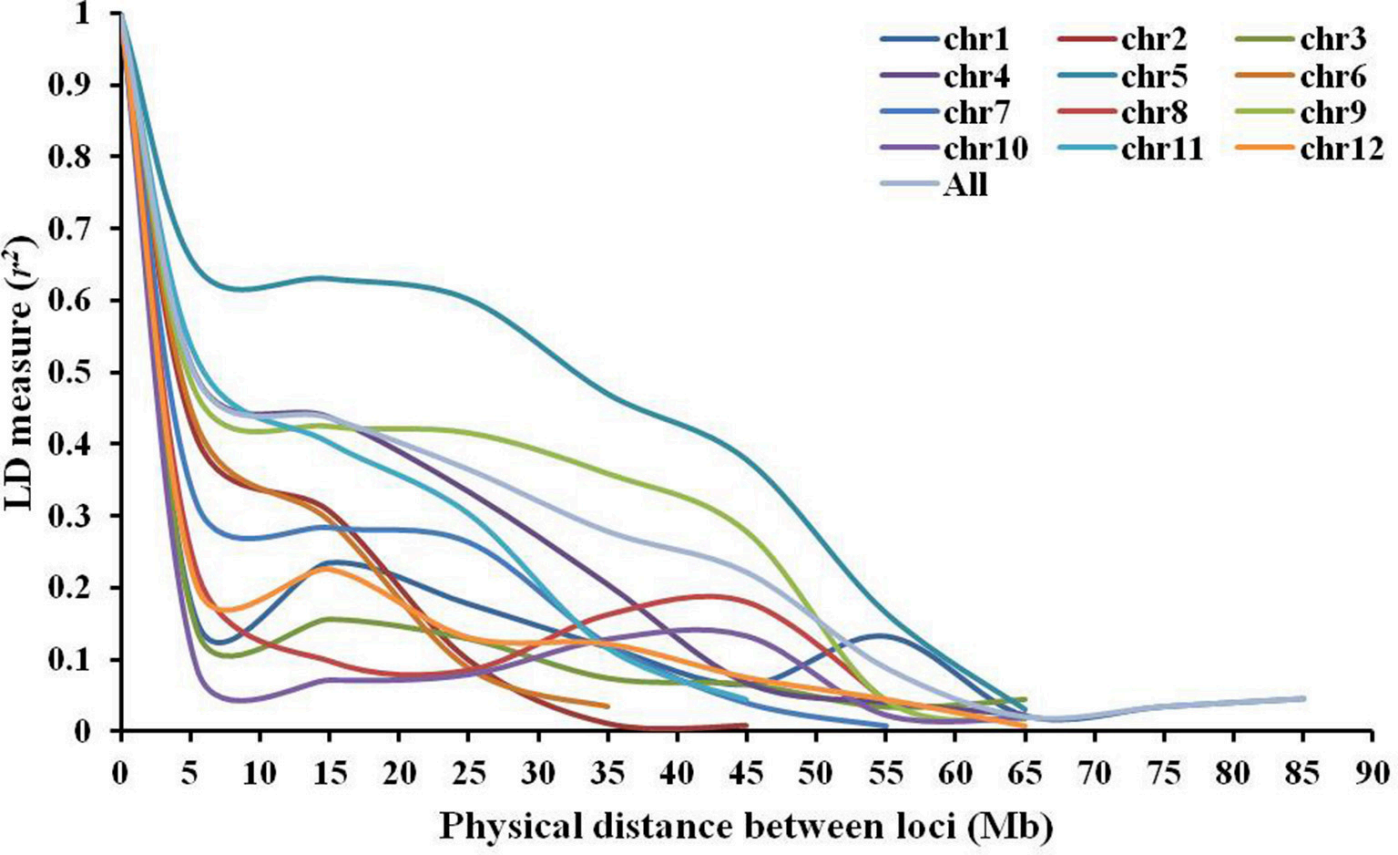
Linkage disequilibrium decay across all 12 chromosomes within the association mapping panel of 164 *Solanum lycopersicum* accessions revealed by plots of linkage disequilibrium (LD) values (*r*^2^) against physical genetic distance (Mb) between pairs of InDel markers.

A mean *r*^2^ of 0.393 was observed between all pairs of linked loci throughout the genome, with a mean maximum *r*^2^-value of 0.571 on chromosome 5 and a mean minimum *r*^2^-value of 0.086 on chromosome 10 (Table 2). The rate of LD decay was different across chromosomes (Figure 5). The baseline *r*^2^-values varied from 0.283 (chromosome 8) to 0.381 (chromosome 10) estimated by the 95th percentile method. Based on LOESS curves, the baseline *r*^2^-values corresponded to physical distances varying from 3.0 to 47.2 Mb. LD decayed within 6.5 Mb on chromosomes 1, 2, 3, 7, 8, 10, and 12, while chromosome 5 showed the lowest decay with a baseline *r*^2^-value of 0.335 reached ~47.2 Mb.

**Table 2:** Summary of linkage disequilibrium (LD) analysis.

**Chromosome**	**No. marker pairs**	***r*^2^ estimates**				**LD decay (Mb)**
		**Median**	**Mean**	**St. Dev**.	**95% percentile**	
1	12,866	0.008	0.156	0.081	0.359	3.2
2	21,501	0.010	0.277	0.098	0.368	6.5
3	13,450	0.008	0.112	0.062	0.359	3.2
4	37,357	0.020	0.357	0.141	0.337	24.5
5	43,986	0.011	0.571	0.205	0.335	47.2
6	29,774	0.012	0.308	0.105	0.331	12.5
7	10,039	0.009	0.224	0.067	0.353	4.8
8	8,838	0.005	0.144	0.055	0.283	4.5
9	27,333	0.012	0.384	0.118	0.313	42.5
10	9,973	0.010	0.086	0.060	0.381	3.0
11	20,063	0.010	0.370	0.116	0.314	24.0
12	13,451	0.014	0.167	0.061	0.319	3.9
All	131,381	0.012	0.393	0.169	0.333	28.5

### Association identification of loci conferring tomato fruit traits

Using the MLM *K* + Q model and Bonferroni correction for *p*-values, a total of 102 genotype/phenotype associations with phenotypic variation explained >2.0% were detected under the threshold *p*-values of 1.30 × 10^−4^ for SSC, 1.19 × 10^−4^ for FW and FSI, 1.22 × 10^−4^ for L^*^ and Chroma, and 1.24 × 10^−4^ for Hue. The numbers of associations per trait ranged from three for Hue to 27 for Chroma. Regions carrying the presumed genes/QTLs were identified on 11 of the 12 chromosomes, but none were detected on chromosome 10 (Figure S2, Table 3). In order to match the associations with previously identified QTL, loci linked to one another within 3 Mb, the lowest LD, on the same chromosome were considered as a unit.

**Table 3:** Markers significantly associated with soluble solid content, fruit weight, fruit shape index, and fruit color parameters (L^*^, Hue, and Chroma) identified by association analysis.

**Trait**	**Marker name**	**Chromosome**	**Physical position (SL2.40)**	***p*-value**	**Phenotypic variation explained (%)**
Soluble solid content	sli2868	1	70500607	1.27E-11	14.5
	sli2875	1	72511190	8.11E-07	20.6
	sli2916	1	81603104	6.53E-06	25.5
	sli311	2	13572187	7.59E-05	13.9
	sli2686	2	25824814	4.55E-07	8.9
	sli2221	4	9883293	8.35E-08	5.6
	sli3316	4	54162751	3.29E-11	13.8
	sli493	5	3635186	2.36E-05	2.6
	sli408	5	14801010	4.11E-06	4.5
	sli500	5	37859546	9.52E-05	3.7
	sli508	5	39790245	3.00E-06	3.6
	sli562	5	54572647	3.65E-06	5.4
	sli571	5	57656536	4.35E-07	10.7
	sli589	5	61241179	7.27E-05	12.2
	sli634	6	7801283	1.60E-07	6.2
	sli660	6	13668472	1.47E-06	9.8
	sli665	6	14558967	4.14E-06	6.3
	sli761	6	37324771	1.37E-14	22.3
	sli919	7	38991134	1.71E-07	15.3
	sli1147	8	31663632	8.68E-06	24.9
	sli4003	9	13623231	4.85E-05	16.5
	sli1324	9	14404856	2.21E-05	4.6
	sli1490	9	15826796	1.95E-08	10.4
	sli1368	9	31114697	1.12E-06	18.4
Fruit weight	sli201	1	29372090	1.63E-05	5.2
	sli2887	1	75604440	2.95E-08	20.5
	sli2438	3	2044312	1.45E-05	17.9
	sli2221	4	9883293	3.26E-12	4.5
	sli3003	4	16716471	2.56E-05	2.1
	sli2307	4	33951126	3.36E-05	2.8
	sli2329	4	41110769	3.36E-05	2.8
	sli2388	4	55504498	4.73E-05	6.5
	sli580	5	59621517	8.94E-05	2.9
	sli1146	8	31471959	7.76E-14	22.9
	sli1147	8	31663632	3.15E-07	12.6
	sli1497	9	20876005	2.61E-15	3.4
	sli1498	9	22109103	7.22E-06	10.5
	sli1516	9	39903394	1.25E-08	5.8
	sli1464	9	65041065	1.28E-09	18.9
	sli1873	11	33962309	5.13E-06	9.2
	sli1886	11	37929907	6.86E-21	7.9
Fruit shape index	sli2653	3	60117959	2.17E-05	3.5
	sli2655	3	60656932	2.31E-06	2.0
	sli2216	4	7766862	2.61E-05	7.4
	sli2347	4	46429902	4.04E-08	2.0
	sli2378	4	53631402	2.68E-07	2.5
	sli2384	4	54772171	1.23E-08	5.2
	sli2419	4	62034593	1.03E-04	2.9
	sli45	5	8155946	1.07E-05	5.4
	sli451	5	23416656	1.92E-08	5.1
	sli470	5	29201547	8.39E-05	5.6
	sli565	5	55625013	2.63E-05	5.0
	sli571	5	57656536	9.59E-05	8.1
	sli665	6	14558967	4.63E-05	3.3
	sli855	7	21689257	4.96E-05	4.2
	sli1414	9	53917837	2.81E-13	2.4
L^*^	sli2799	1	43606482	2.71E-09	5.9
	sli2940	1	87328257	4.05E-11	23.4
	sli2556	3	33299542	8.81E-05	5.1
	sli2215	4	7234645	4.12E-05	5.1
	sli2272	4	25200675	9.71E-06	2.0
	sli2299	4	32139951	1.03E-06	2.6
	sli2302	4	32770761	1.03E-06	2.6
	sli2323	4	39113326	8.79E-07	3.6
	sli2333	4	42413320	1.03E-06	2.6
	sli477	5	30794762	2.76E-14	3.0
	sli495	5	36780476	2.40E-09	2.9
	sli508	5	39790245	2.18E-06	3.1
	sli571	5	57656536	4.10E-07	6.1
	sli685	6	20250580	4.80E-14	14.7
	sli1346	9	23125992	1.42E-08	3.7
	sli1970	12	10754442	2.34E-06	9.2
Hue	sli2792	2	48225893	7.92E-14	3.2
	sli2309	4	34621455	7.81E-05	2.2
	sli685	6	20250580	7.90E-13	4.2
Chroma	sli2799	1	43606482	8.80E-07	9.2
	sli2940	1	87328257	5.00E-09	10.3
	sli296	2	9872987	2.89E-10	11.0
	sli325	2	16913369	2.89E-10	11.0
	sli2792	2	48225893	1.40E-14	5.9
	sli3313	4	53694137	1.19E-05	14.3
	sli3316	4	54162751	1.52E-11	14.6
	sli45	5	8155946	1.17E-25	4.8
	sli418	5	16965721	4.47E-08	3.5
	sli477	5	30794762	8.51E-05	8.0
	sli508	5	39790245	1.77E-05	8.2
	sli522	5	43463720	1.33E-08	7.9
	sli556	5	52455728	7.35E-06	8.5
	sli568	5	56274851	1.33E-05	5.9
	sli571	5	57656536	6.72E-19	17.0
	sli588	5	61020978	5.16E-14	26.3
	sli652	6	12252957	1.08E-06	14.3
	sli685	6	20250580	1.80E-08	16.6
	sli28	6	39518119	1.08E-06	7.5
	sli1322	9	14058288	2.82E-06	9.8
	sli1324	9	14404856	1.64E-06	6.7
	sli1346	9	23125992	9.95E-21	8.7
	sli1520	9	41701481	1.51E-09	11.6
	sli1423	9	56541008	9.77E-13	6.7
	sli1864	11	30139161	3.77E-05	7.6
	sli1970	12	10754442	2.02E-05	11.5
	sli2056	12	38855927	7.97E-09	9.7

Of the 24 genotype/phenotype associations identified for SSC, the phenotypic variation explained by each marker varied from 2.6 to 25.5% (Table 3). Based on the physical distance between markers, at least 19 loci were detected on eight chromosomes and 13 of them had the phenotypic contribution >10%. The 17 markers associated with FW could be assigned to 15 chromosomal regions on seven chromosomes and explained 2.1–22.9% of the phenotypic variation. Five loci on four chromosomes had the phenotypic contribution >10%. Of the 15 associations between FSI and markers detected at 12 chromosomal regions on six chromosomes, the phenotypic variation explained by each marker ranged from 2.0 to 8.1%.

A total of 46 genotype/phenotype associations were detected for three parameters of fruit color, of which 16, 3, and 27 were for L^*^, Hue, and Chroma, respectively (Table 3). These associations were on nine of the 12 chromosomes except chromosomes 7, 8, and 10. The phenotypic variation explained by these markers ranged from 2.0 to 23.4% for L^*^, 2.2–4.2% for Hue, and 3.5–26.3% for Chroma. One marker Sli685 on chromosome 6 was common for all three parameters, seven markers (Sli2799, Sli2940, Sli477, Sli508, Sli571, Sli1346, and Sli1970) were common for L^*^ and Chroma, and one marker Sli2792 on chromosome 2 was common for Hue and Chroma.

Co-localization of QTLs was also observed (Table 3). Seven phenotype/genotype associations for SSC also contributed to FW, fruit shape or fruit color. One QTL on chromosome 5 for fruit shape conferred to Chroma as well. For the three parameters for fruit color, it was not surprised that the QTLs for Hue and Chroma were co-localized because the value of Hue was derived from Chroma (Yang et al., 2004). However, eight QTLs for L^*^ also had impact to Chroma, which was consisted with that the increase of chromaticity makes a color becomes more intense (Yang et al., 2014).

### Identification of loci for SSC and FW in F_2_ populations

To identify loci for FW and SSC in the F_2_ populations, the F_2_ population of OH88119 × Black cherry grown in the spring season 2013 was subjected to initial test using 56 InDel markers. Four were significantly (*p* < 0.05) associated with FW (Table 4). The alleles from Black cherry contributed to small fruit. Two markers Sli2788 and Sli2772 on chromosome 2 contributed 24.2 and 17.9% of total phenotypic variation for FW, respectively. One marker Sli2377 on chromosome 4 explained 6.1% phenotypic variation of FW was close to the marker Sli2388 explaining 6.5% phenotypic variation in the 192 association panel. These four marker-trait associations were validated in the F_2_ population of OH88119 × Black cherry grown in fall 2012. However, only the two markers on chromosome 2 could be validated in the F_2_ population of OH9242 × Black cherry (Table 4).

**Table 4:** Markers significantly (*P* < 0.05) associated with fruit weight (FW) and soluble solid content (SSC) identified in F_2_ populations.

**Trait**	***F*_2_ population**	**Season**	**Marker name**	**Chromosome**	**Physical position (SL2.40)**	***p***	***R*^2^**	**Effect of Black cherry allele**
Fruit weight	OH88119 × Black cherry	Spring 2013	Sli2788	2	47404370	< 0.0001	0.242	−
			Sli2772	2	44259569	< 0.0001	0.179	−
			Sli2377	4	53360768	< 0.0001	0.061	−
			Sli1926	11	53303453	0.0002	0.022	−
	OH88119 × Black cherry	Fall 2012	Sli2788	2	47404370	< 0.0001	0.142	−
			Sli2772	2	44259569	< 0.0001	0.156	−
			Sli2377	4	53360768	0.0034	0.023	−
			Sli1926	11	53303453	0.0003	0.033	−
	OH9242 × Black cherry	Spring 2013	Sli2788	2	47404370	< 0.0001	0.079	−
			Sli2772	2	44259569	< 0.0001	0.077	−
			Sli2377	4	53360768	0.055	0.021	−
			Sli1926	11	53303453	0.4005	0.007	−
Soluble solid content	OH88119 × Black cherry	Spring 2013	Sli2184	4	808145	0.0366	0.055	+
			Sli2416	4	61406781	0.0315	0.058	+
			Sli742	6	33534324	0.0002	0.139	+
			Sli743	6	33733158	< 0.0001	0.149	+
			Sli744	6	34024097	0.0001	0.139	+
			Sli745	6	34110927	0.001	0.118	+
			Sli762	6	37511917	0.0003	0.131	−
			Sli775	6	40230850	0.0011	0.112	+
			Sli1003	7	58815972	0.0039	0.092	+
			Sli1290	9	3428268	0.0047	0.087	+
			Sli1926	11	53303453	0.0106	0.079	−
			Sli1958	12	6425452	0.0233	0.062	+
			Sli2009	12	20925959	0.0401	0.053	+
			Sli2112	12	55874351	0.0389	0.054	+

A total of 14 markers were identified to be significantly (*p* < 0.05) associated with SSC in the F_2_ population of OH88119 × Black cherry grown in the spring season 2013 (Table 4). These markers were from 10 regions on six chromosomes and contributed 5.3–14.9% of total phenotypic variation. Alleles of most markers from Black cherry contributed to high SSC. However, alleles of the marker Sli762 on chromosome 6 and the marker Sli1926 on chromosome 11 from Black cherry contributed to low SSC. Six markers spanning ~6.7 Mb region on chromosome 6 explained the highest phenotypic variation (11.2–14.9%) was in the same region of the marker Sli761 detecting high association with SSC in the 192 association panel.

## Discussion

The LD in cultivated tomatoes has been investigated using various molecular markers and different collections of germplasm. Analyzing a set of 94 cultivars for commercial greenhouse production in Europe with 887 AFLP markers indicates that the LD decay is 15–20 cM (van Berloo et al., 2008), while analyzing 24 fresh market varieties and 39 processing varieties using 434 PCR-based markers shows the LD decay is 6–14 cM within processing cultivars and 3–16 cM within fresh-market cultivars (Robbins et al., 2011). Furthermore, the rate of LD decay depends on chromosomes and tomato types. Processing varieties have greater LD on chromosomes 1, 2, and 5, while fresh-market cultivars have higher LD on chromosomes 6 and 9 (Robbins et al., 2011). Re-sequencing genomes of 360 accessions reveals that the LD decay with physical distance between SNPs occurred at 8.8 kb in *S. pimpinellifolium*, 256.8 kb in *S. lycopersicum* var. *cerasiforme*, and 865.7 kb in *S. lycopersicum* accessions (Lin et al., 2014). A recent study analyzing 300 tomato accessions with 11000 SNPs suggests that the LD decay ranges from 0.2 cM (73 kb) to 49 cM (47 Mb) at chromosomal level (Bauchet et al., 2017). In this study, the LD decay range from 3.0 to 47.2 Mb on 12 chromosomes with an overall of 28.5 Mb in the whole genome, which is larger than previous reports. Molecular marker types, calculation methods and types of tomato accessions could contribute to this difference. SNPs marker data (Lin et al., 2014) provides a smaller LD decay than PCR-based marker data (van Berloo et al., 2008; Robbins et al., 2011), which suggests that high density markers might provide more accurate calculation of LD decay. In addition, previous studies calculated LD decay separately for each market type or species of tomato, while we combined tomato accessions from *S. lycopersicum* var. *cerasiforme* and *S. lycopersicum* to calculate LD decay for each chromosome. The rates of LD decay on seven chromosomes were <6.5 Mb, ~8.7 cM based on the estimation of 1 cM equals ~750 kb (Tanksley et al., 1992), which is consistent with previous data (van Berloo et al., 2008; Robbins et al., 2011). The remaining five chromosomes 4, 5, 6, 9, and 11 with relatively large LD could be biased by chromosome fragments that have been introgressed from wild species in the past decades (van Berloo et al., 2008; Bauchet et al., 2017), e.g., fragments containing resistance genes on chromosomes 5, 9, and 11. However, all studies suggest that the LD is strong in tomato than in other species and association mapping is theoretically feasible with a small number of markers (van Berloo et al., 2008; Robbins et al., 2011; Lin et al., 2014).

Hundreds of genes/QTLs for fruit traits have been detected in tomato using both classical genetic analysis and association mapping. However, due to the lack of direct comparison between genes/QTLs identified by association mapping and classical genetic analysis, only known genes (*fas, y*) or /QTL (*fw2.2*) can be validated through association analysis approach (Shirasawa et al., 2013; Lin et al., 2014). In the present study, a total of 102 phenotype/genotype associations for FW, SSC, fruit shape, and fruit color were detected in the association mapping panel consisted of 192 tomato accessions. Two markers were located within the known gene regions. The marker Sli28 associated with Chroma was at the *og*^*c*^ region on chromosome 6 and the marker Sli2799 associated with L^*^ and Chroma was at the *hp2* region of chromosome 1. The *og*^*c*^ gene can increase the accumulation of lycopene while the *hp2* gene is responsible for more deep pigment in tomato fruits (Mustilli et al., 1999; Ronen et al., 2000). The marker Sli3313 associated with Chroma was at the previously identified QTL region on chromosome 4 (Yang et al., 2004). In their study, the marker LEOH37 explains 21.6% of phenotypic variation of Chroma in the F_2_ population of OH8245 × OH2349 consisted of 160 individuals, while the marker Sli3313 identified in the current study contributed to 14.3% of phenotypic variation in the association mapping panel. No other cloned genes for FW, fruit shape or size, and color were detected. This could be due to several reasons. First, the lack of detection of *ovate, sun, gf*, *t*, and *del* genes was due to only few accessions for each gene were included in the association mapping panel. Second, since the phenotypes of yellow and pink colors were out of our objectives, we did not record these traits. Thus, it was reasonable for not detecting phenotype/genotype associations for yellow and pink colors conditioned by the *r* and *y* genes, respectively. Third, we used a relatively high stringency for association analysis in this study. The *p*-values of 1.19–1.30 × 10^−4^ used here were much lower than 0.005 used in other studies (Xu et al., 2013; Zhang et al., 2016; Bauchet et al., 2017), which could decrease the power of association analysis. Fourth, it has been reported that the genome of cherry tomato accessions is a mosaic composed of polymorphisms of *S. pimpinellifolium* and *S. lycopersicum* (Blanca et al., 2015), which might also cause the failure of association mapping.

Among the 102 phenotype/genotype associations for FW, SSC, fruit shape, and fruit color, 22 with the *p* < 1E-10 were considered as the strongest associations. By searching the tomato genome annotation database (ITAG-cDNA 3.1, http://solgenomics.net), the numbers of predicted genes within 2 Mb regions corresponding to the 22 markers varied from 19 to 286, of which 615 for SSC, 102 for FW, 41 for FSI, 430 for L^*^, 1,000 for Chroma, and 342 for Hue (Table S5). A lot of predicted genes can be considered as candidate genes for each trait based on their predicted roles in biological process, cellular component, and molecular function. For examples, Solyc04g018020, Solyc04g018030, and Solyc09g031560 are putative serine/threonine-protein phosphatase genes. Solyc04g018063 is a putative cytochrome P450 gene. Solyc04g018147 is a putative DnaJ heat shock amino-terminal domain protein gene. Solyc09g030420 is a putative auxin response factor gene. Solyc11g04555 is a putative Myb domain protein gene. All these kinds of genes are considered as candidates for FW (Huang and van der Knaap, 2011). The use of structural populations for genetic analysis results in identification many loci conferring fruit traits in tomato. However, only 28 loci for FW and 11 loci for fruit shape can be detected in at least two independent studies (Grandillo et al., 1999). To identify and validate real QTLs conferring FW, we made crosses between the small fruited tomato accession Black cherry and two medium-sized tomato accessions OH88119 and OH9242 to develop F_2_ populations. The OH88119 × Black cherry F_2_ was divided into two sub-populations grown in two seasons. Three QTLs for FW were identified from the F_2_ sub-population of OH88119 × Black cherry grown in the spring season of 2013 and validated in the F_2_ sub-population of the same cross, but two of them could be detected in the F_2_ population of OH9242 × Black cherry. This suggests that the QTL for FW is affected by genetic background. Two markers Sli2788 and Sli2772 on chromosome 2 contributed 24.2 and 17.9% of total phenotypic variation, respectively, were at each side of *fw2.2* region (Frary et al., 2000). The marker Sli1926 on chromosome 11 contributing 2.2% phenotypic variation located at the known locus *fw11.3* region (Huang and van der Knaap, 2011). This result suggests that both *fw2.2* and *fw1.3* contribute small fruit in Black cherry, which is not in our interest. The marker Sli2377 was significantly associated with FW in both F_2_ sub-populations of OH88119 × Black cherry and marginally (*p* = 0.055) associated with FW in the F_2_ population of OH9242 × Black cherry. The physical distance between Sli2377 and Sli2388, a marker detected significant association with FW in the association mapping panel but not polymorphic among OH88119, OH9242 and Black cherry, was 2.14 Mb that was smaller than the LD on chromosome 4. Thus, this region could be one unit conferring FW. Comparing the physical positions of markers (Grandillo et al., 1999) linked to known loci for FW on chromosome 4, there is no known loci between markers Sli2377 and Sli2388. Therefore, this locus could be a novel one for FW.

In conclusion, association mapping using InDel marker data was applied to uncover the genomic regions harboring genes underlying FW, SSC, shape, and color in tomato fruits followed by confirmation with F_2_ populations in this study. The results demonstrated that the use of limited number of InDel markers and a relatively small number of accessions was effective in validating known genes/QTLs and identifying novel genotype/phenotype associations for marker-assisted selection of fruit traits in tomato.

## Author contributions

Conceived and designed the experiments: HS, WY. Performed the experiments: XL, XG, HZ. Analyzed the data: XL, XG, WY. Contributed reagents/materials/analysis tools: HS, WY. Wrote the paper: XL, XG, WY.

### Conflict of interest statement

The authors declare that the research was conducted in the absence of any commercial or financial relationships that could be construed as a potential conflict of interest.

## Supplementary material

Figure S1 Distribution of phenotypic data in 192 tomato accessions.

Figure S2 Physical map of InDels and QTLs for fruit weight (FW), soluble solid content (SSC), fruit shape index (FSI), and color parameters (L^*^, Hue, and Chroma) on tomato chromosomes. The relative map position of each InDel can be found in Table S2.

Table S1 Description of 192 tomato accessions used in this study.

Table S2 Chromosomal position, primer sequence, number of alleles, and polymorphic information content (PIC) for markers used in this study.

Table S3 Information of the 56 InDel markers used for genotyping the F_2_ populations.

Table S4 Analysis of variance for soluble solid content (SSC), fruit weight (FW), sruit shape index (FSI), fruit color parameters (L, Hue, and Chroma) in 192 tomato accessions grown in 2013 and 2014.

Table S5 Annotated genes in 2 cM region around the markers associated with traits with *e* < 1E^−10^.
