# Supplementary figures and images for: Association and Genetic Identification of Loci for Four Fruit Traits in Tomato Using InDel Markers
Source: Front Plant Sci. 2017 Jul 19;8:1269. doi: 10.3389/fpls.2017.01269 (PMC5515879; doi:10.3389/fpls.2017.01269)

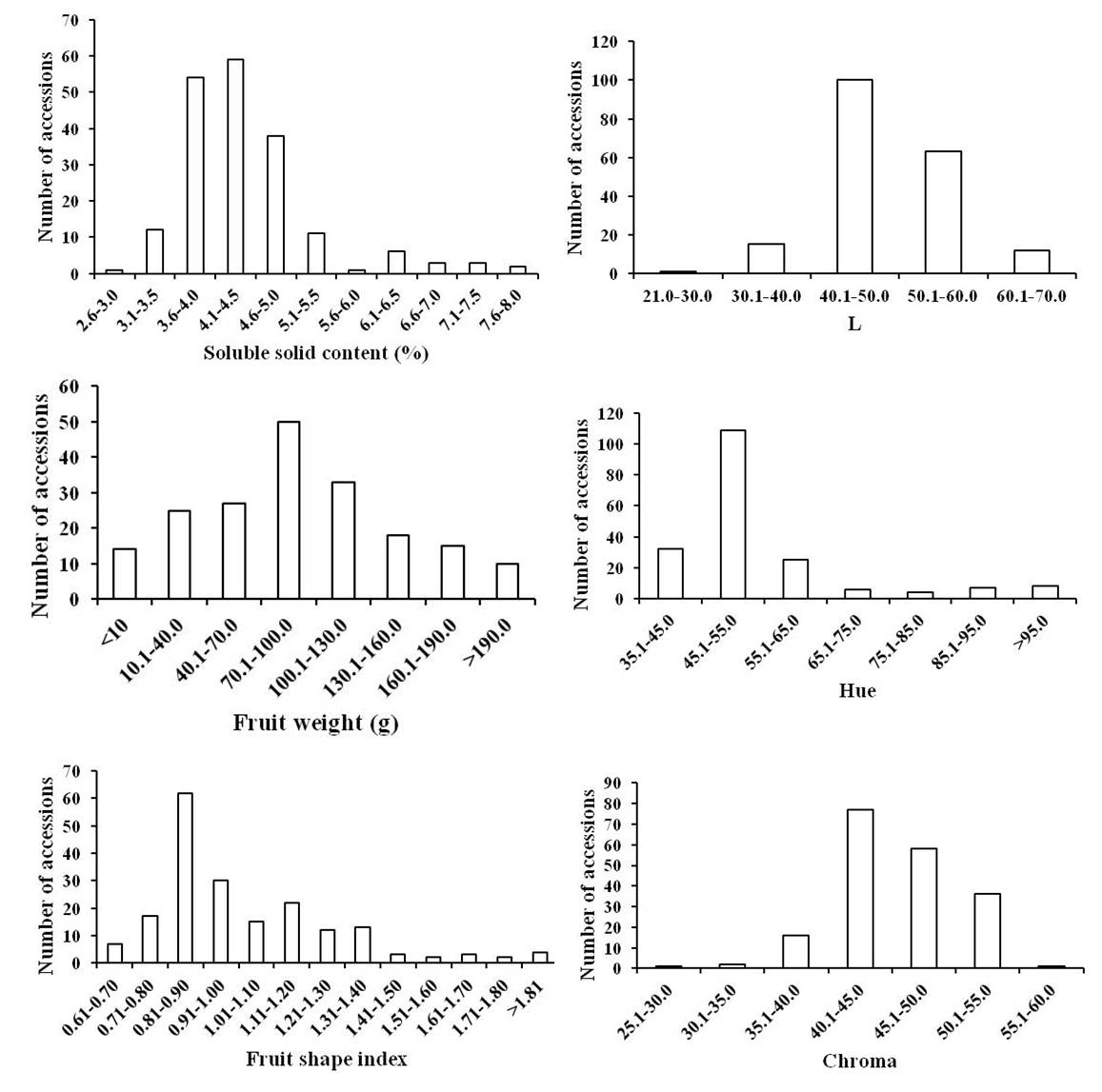

Supplement: Figure S1 — Distribution of phenotypic data in 192 tomato accessions. [file Image1.jpeg]

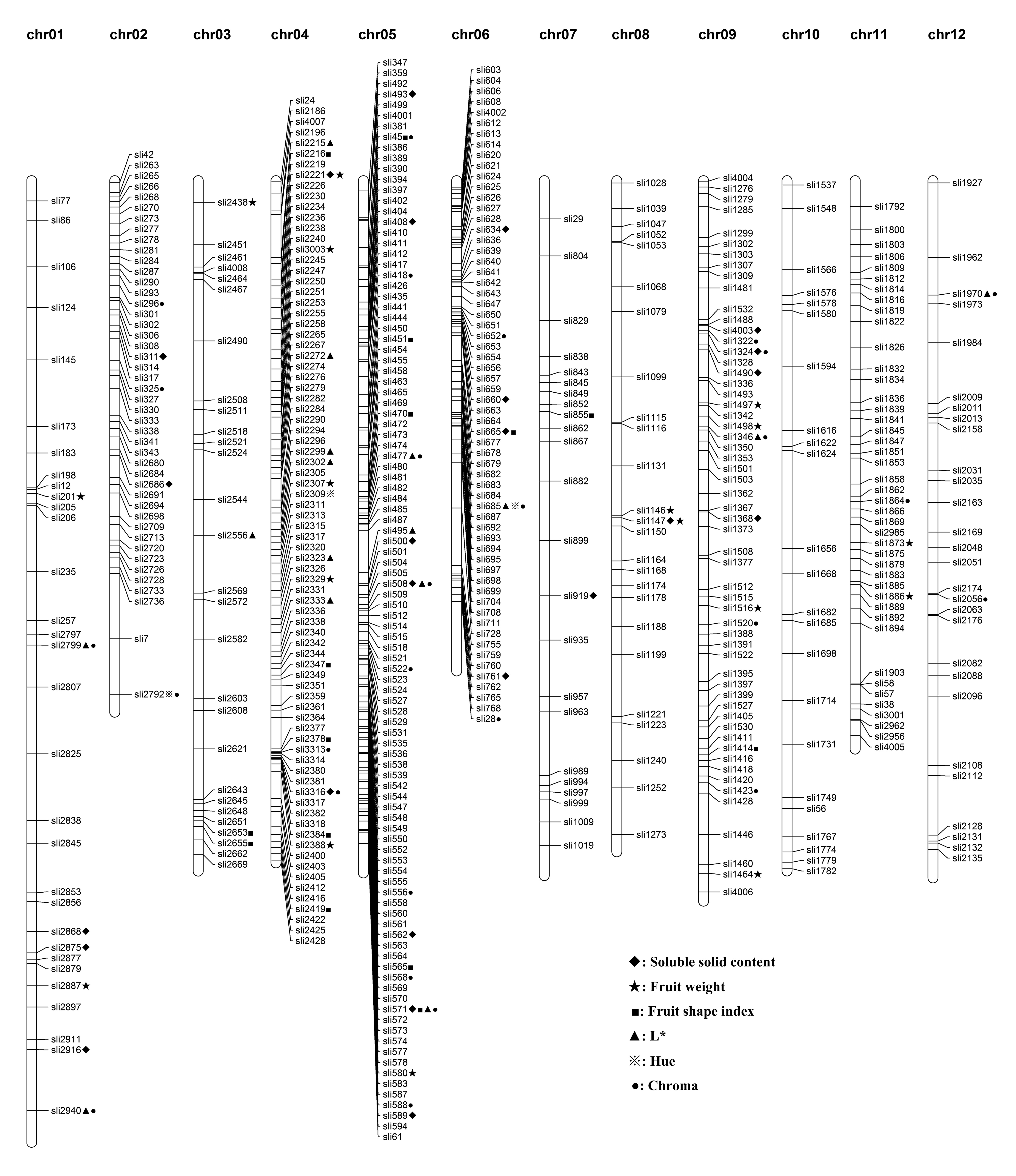

Supplement: Figure S2 — Physical map of InDels and QTLs for fruit weight (FW), soluble solid content (SSC), fruit shape index (FSI), and color parameters (L*, Hue, and Chroma) on tomato chromosomes. The relative map position of each InDel can be found in Table S2. [file Image2.jpeg]
